# Development and validation of deep learning algorithms for automated eye laterality detection with anterior segment photography

**DOI:** 10.1038/s41598-020-79809-7

**Published:** 2021-01-12

**Authors:** Ce Zheng, Xiaolin Xie, Zhilei Wang, Wen Li, Jili Chen, Tong Qiao, Zhuyun Qian, Hui Liu, Jianheng Liang, Xu Chen

**Affiliations:** 1 Department of Ophthalmology, Xinhua Hospital Affiliated to Shanghai Jiao Tong University School of Medicine, Shanghai, China; 2 Joint Shantou International Eye Center of Shantou University and the Chinese University of Hong Kong, Shantou University Medical College, Shantou, Guangdong China; 3 Department of Ophthalmology, Shanghai Children’s Hospital, Shanghai Jiao Tong University, Shanghai, China; 4 Department of Ophthalmology, Shibei Hospital, Shanghai, China; 5 Department of Ophthalmology, Shanghai Aier Eye Hospital, No. 1286, Hongqiao Road, Changning District, Shanghai, 200050 China; 6 Aier School of Ophthalmology, Central South University, Changsha, Hunan Province China

**Keywords:** Biotechnology, Computational biology and bioinformatics, Mathematics and computing

## Abstract

This paper aimed to develop and validate a deep learning (DL) model for automated detection of the laterality of the eye on anterior segment photographs. Anterior segment photographs for training a DL model were collected with the Scheimpflug anterior segment analyzer. We applied transfer learning and fine-tuning of pre-trained deep convolutional neural networks (InceptionV3, VGG16, MobileNetV2) to develop DL models for determining the eye laterality. Testing datasets, from Scheimpflug and slit-lamp digital camera photography, were employed to test the DL model, and the results were compared with a classification performed by human experts. The performance of the DL model was evaluated by accuracy, sensitivity, specificity, operating characteristic curves, and corresponding area under the curve values. A total of 14,468 photographs were collected for the development of DL models. After training for 100 epochs, the DL models of the InceptionV3 mode achieved the area under the receiver operating characteristic curve of 0.998 (with 95% CI 0.924–0.958) for detecting eye laterality. In the external testing dataset (76 primary gaze photographs taken by a digital camera), the DL model achieves an accuracy of 96.1% (95% CI 91.7%–100%), which is better than an accuracy of 72.3% (95% CI 62.2%–82.4%), 82.8% (95% CI 78.7%–86.9%) and 86.8% (95% CI 82.5%–91.1%) achieved by human graders. Our study demonstrated that this high-performing DL model can be used for automated labeling for the laterality of eyes. Our DL model is useful for managing a large volume of the anterior segment images with a slit-lamp camera in the clinical setting.

## Introduction

Anterior segment imaging has largely improved documentation and understanding of anterior segment structures and pathological processes. With the rapid advancement of deep learning (DL) in healthcare, it is now possible to perform automated detection of several anterior segment eye diseases, such as pterygium^1^, corneal ulcer^2^, and cataracts using anterior segment photographs^3^. There is an increasing need for evaluation of corneal, glaucoma, and lens disease on DL algorithms with anterior segment imagery in telemedicine approach or clinical practice^4–6^.

Due to the different types and stages of ocular diseases that are often present in the right and left eyes, it is critical to identify the eye at the laterality level to evaluate the disease characteristics. In previous studies, several authors have proposed automated labeling of the eye laterality on the color fundus photograph using DL models with promising results^7–9^. Compared to fundus photos, it is more challenging and time-consuming for an investigator to identify the eye side without automatic eye side labeling in anterior segment photography.

In this study, we developed a DL system to automatically distinguish the sides of the eyes (left and right sides) from anterior images that were captured by the Scheimpflug camera. We also investigate the performance of this model to predict eye laterality in photographs taken by a slit-lamp digital camera in the clinical setting.

## Methods

The study was conducted in accordance with the tenets of the Declaration of Helsinki and approved by the Institutional Review Board of Shanghai Aier Eye Hospital (IRB: SHAIER2020IRB10). Informed consent was waived because of the retrospective nature of the fully anonymized images.

### Image datasets

In this cross-sectional study, anterior segment photographs recorded with a Pentacam HR (Oculus Optikgerate GmbH, Wetzlar, Germany) were extracted from clinical databases at Shanghai Aier Eye Hospital between June 2018 and December 2019. We selected 8,593 subjects, including 5,386 normal subjects [inclusion criteria: (1) refractive errors less than ± 3 diopters, and (2) without other eye diseases]; 1446 subjects with cataracts; 1,201 subjects with dry eyes; and 560 subjects with strabismus. The diagnoses were based on the evaluations of the subjects’ electronic medical records. The eye laterality was labeled by the pre-defined examination sequence (right eye first and then left eye) and then confirmed by the operators in the field during the examination course. All photographs were manually reviewed by a post-graduate medical student (L.H) and reviewed again by the supervising cataract specialist (Z.C) to ensure correct categorization. Only the images with the quality parameter (QS) of the Pentacam HR that were marked "OK" were included in the study.

**Figure 1:**
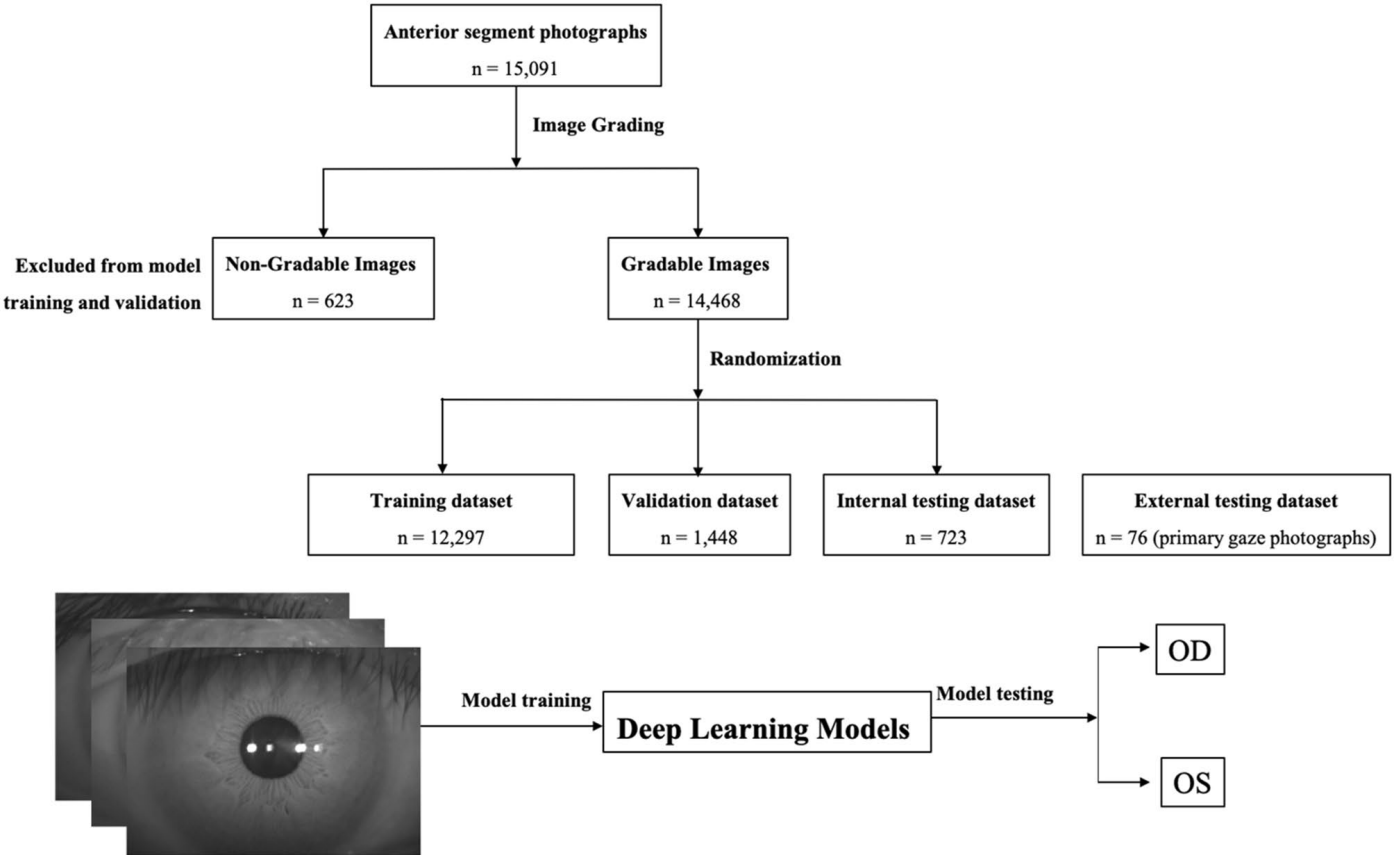
Diagrams that show the grading workflow of anterior segment photographs.

A total of 15,091 photographs were collected. Among these photographs, 623 photos (4.1%) were excluded because of corneal scar, corneal degeneration, pterygium, or any history of eye surgery; 14,468 photographs (7170 right eye photographs and 7298 left eye photographs) remained for the dataset. This dataset was randomly divided into three sets: a training set (85% of dataset, 6094 right eye photographs and 6203 left eye photographs) to develop the DL model; a validation set (10% of dataset, 718 right eye photographs and 730 left eye photographs) to tune the parameters of the trained model and a testing set (5% of dataset, 358 right eye photographs and 365 left eye photographs) to evaluate the performance of the developed model. To test the generalization ability of the DL model, we further assess one external testing set: 76 primary gaze photographs taken of 76 cataract subjects before cataract surgery by a Topcon DC-3 8-megapixel digital camera attachment for the SL-D7 slit lamp (Topcon Inc., Tokyo, Japan). All patient-specific information (e.g., patient identification number, name, date of birth, age, and sex) was removed from the datasets. To simulate eye laterality detection in clinical scenarios, we cropped the original images to remove the media and lateral canthal areas to perform further image preprocessing. Diagrams of the grading workflow of the anterior segment photographs are shown in Fig. 1.

### Development of deep learning (DL) model

In this study, we trained the model by fine-tuning the pre-trained convolutional neural networks (CNNs). The applied CNNs were VGG16 with 23 layers (Visual Geometry Group, Department of Engineering Science, University of Oxford)^10^, Inception-V3 with 159 layers (Google, Inc.)^11,12^, and MobileNetV2 with 88 layers (Google, Inc.)^13^. All CNNs were pretrained on approximately 1.28 million images (1000 object categories) from the 2014 ImageNet Large Scale Visual Recognition Challenge^12^. However, as the recognition task of this work largely differed from that of ImageNet, we further applied transfer learning and fine-tuning of the original CNNs weights for the 2-class eye laterality classification. Further training was performed by initially unfreezing the last 2 layers. The global average pooling layer of the models is then followed by a batch normalization layer, drop-out layer (50%) and 2 dense layers (512 nodes and 1 node). The image pixels were rescaled to values of 0 to 1 and interpolated to fill a 224 × 224 matrix to match existing pre-trained networks. Data augmentation was performed to enhance the dataset, including random horizontal flipping and adjustments to saturation, brightness, and contrast. A minibatch gradient descent of size 32 was employed for training, with an Adam optimizer learning rate of 0.0001 for better convergence. All the DL models and strategies were implemented in the TensorFlow framework (Google; v.2.1.0) with Keras API (v.2.2.4). The models were trained on an Ubuntu 16.04 operating system with Intel Core i7-2700 K 4.6 GHz CPU, 128 GB RAM, and NVIDIA GTX 1080 Ti 12 GB GPU.

### Validation of DL model

To better understand and demonstrate the DL model, we applied the gradient-weighted Class Activation Mapping (CAM) to highlight the area in which the DL model may focus on eye laterality detection. CAM uses the class-specific gradient information that flows into the final convolutional layer of a CNN to produce a coarse localization map of the important regions in the image. This technique is crucial for interpreting network output and validating whether the network learned meaningful features.

Moreover, t-distributed stochastic neighbor embedding (t-SNE) was employed to intuitively analyze the characteristics of the extracted hierarchical features (512 features from InceptionV3 model) from the DL model^14^. t-SNE is a tool for visualizing high-dimensional data, such as the DL features in this study. The tool converts similarities between data points and minimizes the Kullback–Leibler divergence of the joint probabilities between the low-dimensional embedding and the high-dimensional data.

### Human–machine comparisons

We also validated the DL model by performing human–machine comparisons. The external testing dataset (76 primary gaze photographs) was employed to compare the DL models’ referral decisions with the decisions made by human graders. Three ophthalmologists with different levels of clinical experience (L.JH with 1 year, C.XP with 10 years, H.YP with 20 years) who were blinded from the data set collection were instructed to independently make a decision about each testing image. Using CAM that is generated from the external testing dataset, we also reviewed and categorized the misclassified photographs according to the three most commonly viewed features: (1) eyes with coexisting eye conditions, such as involutional ptosis, trichomegaly or misdirected lashes; (2) eyes with other photo conditions, such as overexposure or low exposure and off-center; and (3) other features (incorrect labeled).

### Statistical analysis

All statistical analyses were performed using the Python (version 3.6) and Scikit_learn modules (Anaconda Python, Continuum Analytics). The applied performance metrics included accuracy, sensitivity, and specificity with 2-sided 95% CIs. The formulas for calculating the accuracy, sensitivity, and specificity were defined as

1 $$\mathrm{Accuracy}= \frac{True \, Positive+True \, Negative}{All,}$$

2 $$\mathrm{Sensitivity}=\frac{True \, Positive}{True \, Positive+False \, Negative}$$

3 $$\mathrm{Specificity}=\frac{True \, Negative}{True \, Negative+False \, Positive}$$

where accuracy, sensitivity, and specificity are reported according to the Standards for Reporting of Diagnostic Accuracy Studies (STARD)^15^. The area under the receiver operating characteristic curve (AUC) metrics were utilized to determine the performance of our DL model.

## Results

**Figure 2:**
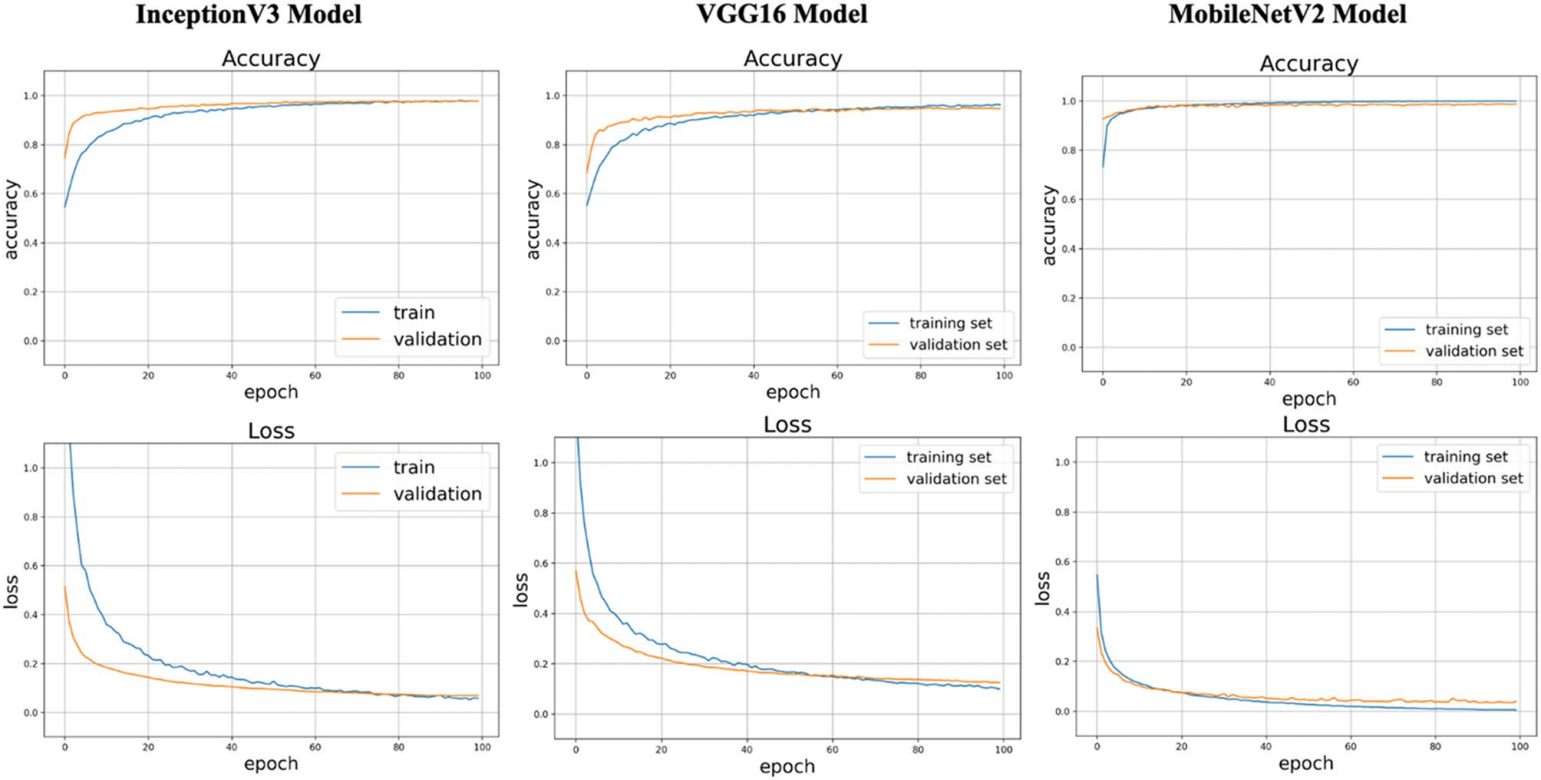
Training curve for the DL model. After training for 100 epochs, our DL model showed no improvement in both accuracy and loss function.

**Figure 3:**
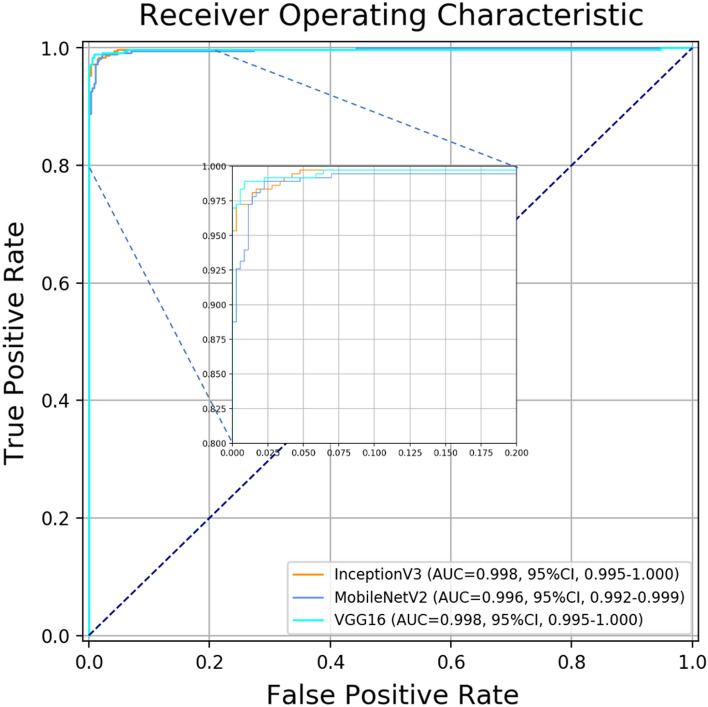
Performance of this DL model in detection of eye laterality in the testing set.

The details of the eye laterality distribution of the datasets are shown in Table 1. After training for 100 epochs (model showed no improvement in both accuracy and loss function), DL models for classifying the right eye versus the left eye achieved an average AUC of 0.998 (95% CI 0.924–0.958) with the InceptionV3 model, an average AUC of 0.998 (95% CI 0.995–1.000) with the VGG16 model, and an average AUC of 0.996 (with 95% CI 0.992–0.999;) with the MobileNetV2 model (Figs. 2 and 3). Considering the similar results, we only selected the InceptionV3 model for further experiments in this study. The grading performance of the DL model compared with three human graders using external testing datasets is shown in Table 2. Compared to the DL model (an accuracy of 96.1% with 95% CI 91.7%–100%), our results showed that 3 human graders had a limited ability to discern the right eye versus the left eye, with an accuracy of 72.3% (95% CI 62.2%–82.4%) for grader 1, 82.8% (95% CI 78.7%–86.9%) for grader 2 and 86.8% (95% CI 82.5%–91.1%) for grader 3.

**Table 1:** The eye laterality distribution of the training, validation and testing datasets.

	Pentacam datasets			Eye Subtotal	Slit-lamp photographs
	Training set	Validation set	Internal testing set		External testing set
Right eye	6094	718	358	7170	38
Left eye	6203	730	365	7298	38
Total	12,297	1448	723	14,468	76

**Table 2:** The grading performance of the DL model comparing with three human graders using external testing datasets.

	Accuracy (95% CI)	Specificity (95% CI)	Sensitivity (95% CI)
**Deep learning model**	0.961 (0.917–1.000)	0.974 (0.938–1.000)	0.947 (0.897–0.997)
**Human graders**			
#1	0.723 (0.622–0.824)	0.750 (0.653–0.847)	0.694 (0.590–0.798)
#2	0.827 (0.742–0.912)	0.767 (0.672–0.862)	0.909 (0.844–0.974)
#3	0.868 (0.792–0.944)	0.872 (0.797–0.947)	0.865 (0.788–0.942)

**Figure 4:**
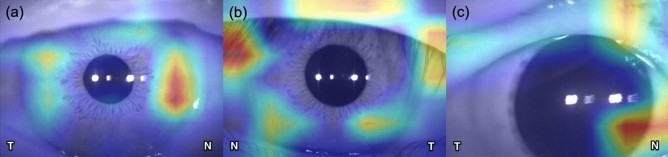
Class activation map was able to identify characteristic areas (nasal limbal area and iris area) in anterior segment photographs, which are presented as a heat map. (*N* nasal, *T* temporal).

CAM was developed during the training phase to determine which regions of the anterior segment photographs were being paid the most attention. Figure 4 shows a CAM that is generated from the InceptionV3 model; note that activation was mostly shown in the nasal limbal and iris area (Fig. 4a).

**Figure 5:**
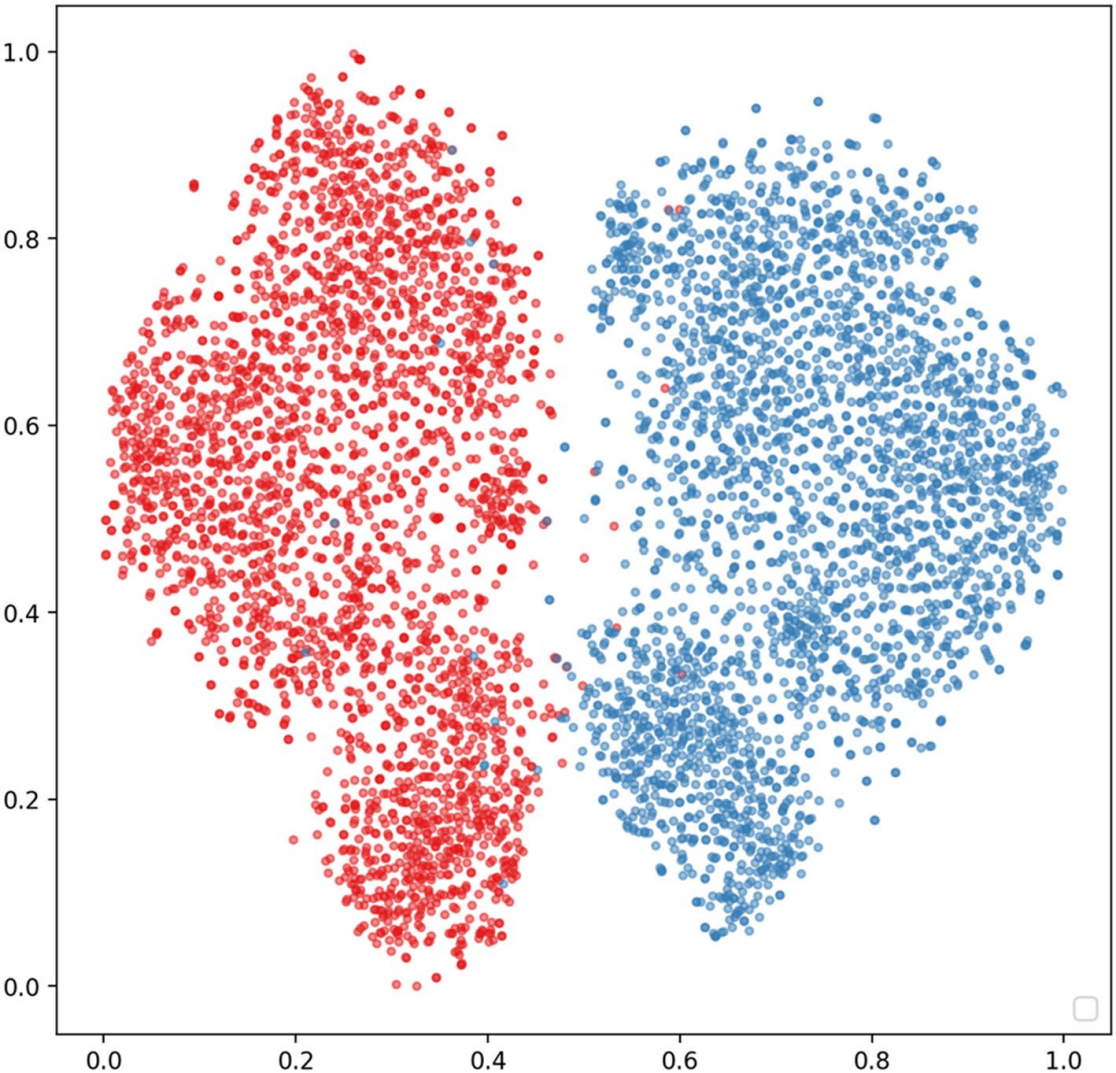
t-SNE plots of features associated to the different types of synthetic images. The red versus blue dots represent hierarchical features extracted from right eye vs. left eye, respectively, of anterior segment photography.

Figure 5 shows the t-SNE visualization for the DL features with red dots versus blue dots to denote the right eye versus left eye, respectively, in the feature space. From the t-SNE visualization, the deep learning features resulted in two objectively separated clusters.

Table 3 shows the reasons for misclassification during human comparisons versus machine comparisons. Based on the CAM generated from the external testing dataset, the most common reason for misclassification was eyes with coexisting eye conditions (n = 17, 58.6%), mainly ptosis (Fig. 4b). Another reason for misclassification consisted of eyes with other conditions for photography (n = 11, 37.9%), mainly overexposure or low exposure and off-center (Fig. 4c).

**Table 3:** The proportion of reasons for misclassification by the human graders.

Reason	No. (%)
With coexisting eye conditions	17 (58.6%)
With other photo conditions	11 (37.9%)
Others	1 (3.4%)

## Discussion

In this study, we developed a robust and highly accurate DL model to distinguish the eye laterality on anterior segment photographs that were captured by the Scheimpflug imaging system. This DL model was also validated by slit-lamp photography, an imaging modality which is commonly employed in daily work. To our knowledge, there is no DL system for identifying the laterality of anterior segment photography. We believe that this DL model is highly reliable in clinical practice for anterior segment images with different pathologies and as a large-scale telemedicine approach without eye laterality labels, especially after the worldwide coronavirus disease 2019 (COVID-19) crisis.

In this study, our DLS module achieved a sensitivity of 94.7% (95% CI 89.7%–99.7%) and a specificity of 97.4% (95% CI 93.8%–100.0%) in eye laterality identification, which is similar to the performance of the algorithm for the fundus laterality detection^7,8^. Even for an experienced ophthalmologist, the accuracy of the eye side labeling is lower in the anterior segment images than labeling in the fundus images. The optic nerve and retinal vessel are significant indicators that are used to identify the laterality of the fundus images, but the cornea, iris, pupil, and even the conjunctival vessel are often similar in both eyes. Previous studies have shown that DL can acquire an additional biomarker or new knowledge from medical images by an optimized algorithm and recognize signals of disease that medical experts may not detect by their experience or knowledge^16,17^. Because the medial and lateral canthus can be the reference of the eye side, we purposely cropped anterior segment images with only the limbus area and part of the eyelids during image preprocessing. Note that the algorithm actually identifies the eye side by activating the nasal iris area in images during CAM analysis. Using 3D swept-source anterior segment optical coherence tomography, Invernizzi et al. reported that the iris’s morphology varied by iris sector and the iris color was associated with differences in iris volume and thickness^18^. Sidhartha et al., determined that the iris thickness was correlated with the iris surface characteristics, such as the color and number of crypts^19^.

DL has made substantial progress in medical image analysis and achieved excellent performance that exceeds traditional diagnosis by human experts. Even though most ophthalmic devices can record the eye laterality in their operation system or display the laterality label on images in related software, the original image and laterality information are separately stored in the databases of these devices. Manual labeling of the eye side in the image dataset is labor-intensive and time-consuming. It is necessary to develop a DL system for the application of eye laterality classification.

The dataset images were initially captured by an infrared (IR) camera with the Pentacam. Black-and-white (B/W) IR images were generally employed in diagnosis and treatment in ophthalmology, such as in the Verion or Callisto eye system, which uses anterior segment IR images to register the iris for guiding toric intraocular lenses alignment. However, these two image guide systems still need to manually match the eye laterality^20^. We tested the DL by using the color photography taken by a slit lamp digital camera. Although the DL algorithm was initially developed from the image dataset collected by the Scheimpflug camera, we proved it also has excellent accuracy with an independent image dataset, where the images were collected from primary gaze photographs by a digital camera. For this dataset, the manual label method had an accuracy that varied from 72.3 to 86.8%, while our module was able to detect eye laterality with an accuracy of 96.1% (95% CI 91.7%–100%). The results showed that the performance of DL was significantly better than that of the manual method, which proved its reliability in anterior segment image management.

One of the key purposes of this study was to validate DL models for automated eye laterality detection. In any screening or telemedicine program, misclassification results create unnecessary referral and burden to the healthcare system. To further validate the performance of the DL model, we also evaluated misclassification of human graders by evaluating the CAM. In this study, note that the DL model not only shows high accuracy but also highlights locations that are informative for human graders, which can potentially serve as a training tool for clinicians.

One limitation of this study was that images were captured in B/W mode, which provided lower information than the color images. It is possible for the DL system to be trained with color and high-resolution photography. However, the DL system required a vast amount of datasets and longer training times, which was impractical for this study. We also consider that our DL system would be more clinically useful because a color photograph can be easily converted to B/W mode for analysis and is more convenient for multiple clinical sets.

Future work will involve more devices for eye laterality determination, such as a surgery recorder, topography, biometer, or mobile phone camera. In busy clinical settings, eye laterality recognition for a patient eye is more important and meaningful than for a healthy eye. Although we included images from subjects with cataracts (a leading cause of blindness in the world^21^), dry eye, and strabismus (a common eye disorder that affects children^22^), further research is warranted amongst different eye diseases, including corneal scar, corneal degeneration, pterygium, etc. In this study, we excluded 623 images from subjects with rare corneal diseases based on the PRISMA diagnostic test accuracy guideline^23^. How to detect eye laterality with rare corneal is still a challenge. Our DL system cannot achieve satisfactory performance in this exclusion dataset [an accuracy of 64.3% (95% CI 53.5%–75.1%)]. Other machine learning techniques, such as few-shot learning^24^, may be helpful to improve the results.

The DL system can be used by clinicians for eye laterality recognition. Further studies are needed to investigate where this technology could be best utilized within clinical and research settings.
